# The Principles and Practice of Surgery

**Published:** 1872-02

**Authors:** 


### Books Reviewed.*

*The Principles and Practice of Surgery.* By John Ashhurst, M.D. 8 vo. p. p. 1011. Philadelphia, H. C. Lea, 1871.

Most practitioners of medicine and surgery actively engaged, particularly those residing in rural districts, require books less given to the free discussion of questions, than to statements respecting the pathology, diagnosis, and treatment of surgical affections " in as concise a manner as may be compatable with clearness, a condensed but comprehensive description of the Modes of Practice now generally employed." (Preface.)

The want of such a work, embracing the latest views in surgery being felt, it is not surprising that many were impatient of the delay in the appearance of this, as well as the work of Dr. Hamilton; and it may be confidently expected that two works on General Surgery, coming from such authority as these do, will fully meet the demands, more particularly of the ordinary practitioner than of him who makes surgery more of a specialty, and who investigates with a scientific purpose.

The general plan of the work before us is similar to that adopted by Holmes, and Erichsen, and is divided only into chapters, XLVII in number, com-

* On account of the press of original matter several books remain until next month for Notice and Review.

mencing with Inflammation, followed by general Operations, Minor Surgery, Amputations, Surgical Injuries, and Surgical Diseases. The author has availed himself of the writings of most of the "Masters of the Profession," and has also combined his own large experience in the Hospital of Philadelphia.

The steps in the process of *Inflammation*, according to the author, after the application of the exciting cause, are respectively ,change of functional activity, (first an increase which may be followed by a diminution); changes of nutrition, *followed by* determination of blood with blood changes, temporary hypertrophy (according to Virchow)—an enlargement of the cells of the affected part, to which most of the swelling is due—nerve changes; and formative changes, consisting in the deposit of lymph, serum and pus. In the production of lymph the views of John Hunter are discarded, and those of Virchow—that of proliferation of pre-existing cells of Cohuheim—of a transfer of the white corpuscles (wandering cells of Recklinghausen), and of Billroth—of the change of the intercellular substance of the connective tissue, are all accepted. The origin of pus cells is spoken of more doubtfully, and allusion is made to the theories of Virchow—proliferation of connective tissue corpuscles of Cohuheim—migration of white corpuscles through the vascular walls, and of Stricker, who acknowledges both sources, but thinks the increase is due to multiplication of the cells themselves.

Our author does not seem to be quite clear in regard to the precise time at which inflammation may be said to be established (although on p. 42 it is stated that all the symptoms should be present in a part in combination before the diagnosis can be properly made.) Thus, change of color (due to hyperæmia) is given as one of the first of the phenomena of the 1st stage; the deposit of lymph takes place in the 2d; and pus in the 3d. On p. 35 it is stated "acute hyperæmia of the mammary gland * * is sometimes with difficulty prevented from running into absolute inflammation." But on the same page, "hyperæmia" is ascribed as being "due to an increased attraction exerted by the tissues of the *inflamed* part" &c.,and on p. 35 and 60 Simon is quoted, who says, "A part **receives more blood because it is *inflamed*." Again, on p. 59 "* The increased quantity of blood * is itself *caused by inflammation*." (Italics ours.) We suppose the author means the functional changes—a diminished vitality—invites the system to respond by sending more blood, instead of the presence of blood being the cause of the remaining steps in the process. Gross emphatically teaches that the deposit of lymph marks the actual occurrence of inflammation. It may be inferred, however, that Vichow [1] believes there is no exudation due to inflammation *per se.*, but deposit occurs from change in the condition of the affected part, though he admits the existence of exudation in inflammation of all *vascular* tissues.

The author does not consider inflammation a "disease" which is to be combated as such, but "a modification of natural processes" to be treated with due regard to the local and general condition of the patient. *Bleeding* is discarded

1 Chance, Ed. p. 436.

as a cure of inflammation, but is useful in mechanically relieving the congestion of the vital part, and calomel and tartar emetic are only admitted for use in the best selected cases. We—like Meigs and Pepper, in the treatment of pneumonitis—"never felt sure it '(calomel)' was of any special service, and of latter years have abandoned it altogether," believing that the profession have already undergone too much rack and torture from former heroism in practice, and which is still taught and followed to some extent. *Opium* is said to be "of all single remedies probably the most useful" by promoting "physiological and functional rest." It may possibly exert a more important influence by arresting in some measure the process of osmosis [2] and cell hypertrophy. Timely support is found not to aggravate the morbid action.

In *Amputations*, Dr. Ashhurst recommends the use of the tourniquet, discarding the view advanced by some that it causes venous thrombosis and resulting pyæmia. After describing the different methods of operating, on p. 103 a short view of the *relative merits* of each is given, summing up as follows:

"If any general rule were to be given, I should say that the circular incision, or Teal's method" (rectangular) 'gives the best stumps in the forearm, the modified circular '(integumentary flap)' in the upper arm and the upper part of the thigh, the common double-flap operation immediately above and below the knee, the circular or lateral-flap in the lower part of the leg, and the oval operation (integumentary) 'at the joints.'" The relative merits of each are not discussed much, but on p. 101 we notice one remark in regard to skin flaps which we are somewhat inclined to question. "Apart from the danger of sloughing, which always attends these long skin flaps, unsupported by muscle, the resulting stump is not so serviceable," &c. Why the integuments are more likely to slough by being rounded into flaps than in the circular method we do not see, unless unequal in length. And in regard to the usefulness of the stump, it is said on p. 105 that most of the tissues change into a fibro-cellular mass, those portions which overlie the extremity of the bone being absorbed. [2] This being true, the value of the stump is about the same in either operation. Since with the muscular flap there is always more drainage and a tedious process of healing, subjecting the patient to greater risk than the circular or one of its modifications. Is it not manifest that the latter class should usually be employed? We think most army surgeons prefer the latter. In no place are skin flaps more desirable than below the knee, making them on either side, a little fuller above than below—not dividing the flap as far back as the cuff is to be skinned off. The Surgeon has five operations at the hip-joint to choose from without any tables showing relative merits. [3] Morton has collected 8 cases in which the operation is stated; of these, 6 were integumentary, and 2 muscular flap operations. Of the former, 4 recovered and 2 died, 1 of which died 12 days after the operation of secondary hœmorrhage;

1 Dis. of Children, p. 183. 2 V. Marshall's Physiology—Note by F. G. Smith, p. 613.
2. See Gross' System of Surgery—Changes of the stump.
3 Amer. Jour. Med Sciences, July 1866, p. 17.

of the latter, 1 recovered and 1 died. These results are much more favorable than in primary operations, but the weight of testimony is in favor of the integumentary operation, Dr. Morton himself entertaining that opinion. The article is concisely and plainly written, but we cannot but wish the author had been a little more explicit in recommending the abdominal tourniquet, and in giving the guides and keys to the articulations.

The chapters on *Injuries in General*, and *Gunshot Wounds*, are admirably handled, and the rules laid down for the treatment of the latter, though briefly discussed, are eminently practical and sound. Excision of the hip in recent gunshot injury is preferred to either amputation or expectancy. We infer, (though it is not clearly expressed) if the case has become secondary, the author thinks amputation affords the better chances for recovery.[1] The All cases of gunshot fractures wounding the knee joint require amputation. In wounds of the ankle joint gouging is admissible. Primary excision of the shoulder and elbow are recommended as preferable to amputation or expectancy, although the death-rate in excision of the latter joint is a little in excess of that in amputation. Excision of the femur is condemned. Excision is faintly assented to in the tibia or fibula separately, and humerus, and recommended in the radius and ulua separately. The author agrees with Mr. Erichsen and Prof. Hamilton in thinking the expectant treatment affords the patient a better chance of recovery in gunshot fractures of the upper third of the thigh, equal with amputation when at the middle, and less than amputation at the lower third. Some, however, think there is but little difference between the lower and upper part of the thigh as regards mortality from operations.[2] Primary operations when required give the patient the best chance, secondary next, and intermediate the last.

Notice is not made of Howard's (of N.Y.,) notion of wiring the ends of the bones together after resection, the treatment being frequently substituted for amputation, but he honors that gentleman by alluding to his treatment of chest wounds (gunshot) by hermetically sealing. The only case known to have recovered was only saved by re-opening the wound. (p. 358, from Circular No. 6, S. G. O.)

The manner in which the authors of impractical ideas are treated is exceedingly lenient throughout the work.

The different methods of arresting hemorrhage are discussed with due regard to each in its appropriate place. On p. 181 it is said "the ligature as now used, * * is when applicable, the very best method of checking arterial hemorrhage." And, on p. 182, "The best material for a ligature is * * ordinary fine whipcord or silk." Also, p. 183, "Metalic ligature thread * * * may properly be applied in cases in which it is desirable to leave the knot in *situ* and close the wound over it, as in certain operations upon the abdominal

1 The authors' views are more fully expressed in the Am. Jour. Med. Sciences for October 1869, p. 466, 467, in a Review of Otis on Excision of the Head of the Femur.

2 See Review of Ashhurst's Ed. of Erichsen's Surgery by Geo. C. Blackman, M.D , in the Am. Jour. Med. Sciences for Jan 1870, p, 172.

cavity: even in these cases, however, it is doubtful if the antiseptic short cut ligature of Prof. Lister would not answer a still better purpose."

*Deligation of Special Arteries* is hurridly passed over. On p. 203 ligation of the common femoral is said to be a "bad" operation, "and the external illiac should be always tied in preference." Some surgeons, both in this country [1] and in Europe, [2] do not hesitate to tie it, though Erichsen speaks boldly against it. The anterior operation for ligation of the primitive illiac only is given.

The chapters on *Fractures* and *Dislocations* in a work of this character, should, in our opinion, be a little more full, for it is a class of injuries with which all physicians have to deal, and all have not access to monographs. In separation of the epiphisis at the upper end of the humerus and fracture of its surgical neck, it is stated on p. 249, "the deformity, which is characteristic, * * consists in the upper end being drawn upwards, inwards, and forwards." Hamilton [3] thinks this is the position of the fragments in most cases, but mentions Dupuytren, Paletta, and others as having seen both fragments pushed outwards. We once heard a case, in a girl 10 years old, of supposed separation of the epiphisis with persistent displaeement of the lower fragment and flattening below the acromion. We notice the omission of the important discovery of Dr. Moore, [4] of Rochester, N.Y., in fracture of the lower end of the radius. Dislocation of the ulna usually co-exists, with displacement of the tendon of the extensor carpi-ulnaris; and until these are restored to position, it is impossible to maintain reduction of the fragment of the fractured bone. A very full and satisfactory account of dislocation of the hip (after Bigelow) is given. The unaided hands of the Surgeon in nearly all cases of fracture and dislocation are sufficient to effect reduction. On p. 227 passive motion of the joints is recommended at the third or fourth week after fracture, and * even then * only with moderation and gentleness." Gross advises passive motion at the end of two weeks, and Hamilton [5] as early as the 7th or 8th day.

In treating *Injuries of the Head*, we think the author has hardly done himself justice, especially on *scalp wounds* in view of their frequency. His views of concussion are peculiar, thinking all except slight cases, are attended with a *bruising* of the brain, and some merge so nearly into compression that the difference cannot be determined—indeed, the conditions are identical. In discussing *trephining*, he treads on disputed soil, is very positive in his advice, and inconclusive in his arguments. We hope to be pardoned if we dissent from the author's views in regard to both *simple*, and *compound depressed fractures*. In both (if impacted in the latter) the operation is opposed even "if symptoms of compression are present." Gross advises the operation in these cases if there is any considerable depression, and while it is difficult to fix the

---

1. Gross' Syst. Surg. vol. 1, p. 945.
2. Holmes Syst. Surg, London Ed.. vol. iii, p. 511.
3. Fractures and Disl , 3d Ed., p. 225.
4. See Med. Record for April 1870, also Hamilton, 4th Ed. Fractures and Dislocations.
5 page 227.

*amount* of depression as a rule for procedure. We should not hesitate to operate in *an adult* in which the depression was an eighth of an inch, even if symptoms of compression were *not* present. The remote consequences of depressed bone are too well known for dispute. Some surgeons of eminent skill would uphold us in operating in a case of *fissure* of the skull, or in case of a blow without fissure, should compression supervene. With children the rule would be different. In traumatic cerebritis calomel and opium are recommended. No allusion is made to Hammond's view—that the bromides,[1] and the oxide of zinc[2] lessen the amount of blood in the brain.

*Injuries of the Spinal Cord and of the Vertebral Column* are discussed with marked interest, and the article shows a thorough acquaintance with the subject; Reduction of displaced vertebræ is advocated when possible.

*Pyœmia* composes an interesting chapter. The author states that the "morbid condition results from the absorption of septic material." Phlebitis is seldom thought to be the cause. Gonorrhæal rheumatism and urethral fever are mild types (p, 429 and 899.

The subject of *Auenrism* is well handled. The author seems less positive in favor of ligature of the femoral in popliteal aueurism than he expresses himself in his edition of Erichsen, being governed by the nature of the case.

In a short chapter on *Orthopœdic Surgery*, the treatment of club-foot is directed to the "mild," and "severe" cases. The latter usually require tenotomy; in regard to the former, while cures may be effected by apparatus, "tenotomy (which has not been proved to do any harm) certainly abbreviates the treatment," (p. 625.) Barwell opposes the operation in all cases. We prefer with Sayre to direct the treatment to cases of *paralysis*, and to those of *contraction* of the opposing tendon without paralysis. In the former class, tenotomy will surely make a useless limb, at least for an indefinite period, but in the latter the operation is required. The differentiation of the two classes can be made when the child is asleep or anæthetized.

The subject of *Urinary Calculus* is well considered, and the various operative procedures well described. We think the author is a little hasty in condemning lithotrity in children under the age of puberty, and unfair in his reference to Guersank's tables. His lithomies number 100 with 14 deaths, and lithrities 40 with 7 deaths, (17½ per cent.) The fact is, 6 of the former died of intercurrent affections, and there remained permanently, 1 rectal and 2 perinæal fistulas, and 3 cases of incontinnence of urine. Of the latter operation 4 died of intercurrent affection, and one in consequence of a fault in the operation, and no bad result occurred in those who survived. Still, no doubt most surgeons will prefer lithotomy in young children.

There are many other points in the book to which we would have referred,

1. N. Y. Med. Jour. for June 1855, p. 203.
2. Diseases of the Nervous System, p. 555.

but we have already exceeded our limits. The work, on the whole, is well arranged, concisely written,—indeed, no more truths could well be put into the same space; and the descriptions are, for the most part, clear. In the author's desire to limit the size of the work, some subjects seem to be rather too hastily passed over; but many of the articles receive due attention and are admirably presented. Among those of especial interest, not already discussed, are those embracing venerial diseases, diseases of the vascular system, excisions, herina, and diseases of the urinary organs. The rules laid down for operative procedures are, in the main, capital, involving late views and inventions; and the whole work gives evidence of having been written by one thoroughly familiar with surgical literature.

We notice but few clerical errors; "patience" is written for *patient*, (p. 757) "coccydynia" for *coccyodynia*, (p. 243.) "Symptoms" is sometimes written when *signs* or *clinical history* would better express the thought. "Attain" is repeatedly used in a wrong sense throughout the book, and a few additional errors and omissions are noticed in an appropriate list.

The wood cuts, many of them, are new and well made, and the book is presented by the publishers in good shape.

